# Individual placement and support (IPS) integrated with specialized substance use disorder treatment: a socioeconomic analysis based on a randomized controlled trial

**DOI:** 10.3389/ijph.2026.1609386

**Published:** 2026-06-25

**Authors:** Kristoffer Andreas Aamodt Andersen, Linn Nathalie Støme, Erlend Marius Aas, Espen Ajo Arnevik, Marianne Riksheim Stavseth, Eline Borger Rognli

**Affiliations:** 1 Department of Psychology, University of Oslo, Oslo, Norway; 2 Section for Clinical Addiction Research, Division of Mental Health and Addiction, Oslo University Hospital, Oslo, Norway; 3 Department of Research and Innovation, Oslo University Hospital, Oslo, Norway

**Keywords:** individual placement and support, integrated care, IPS, socioeconomic analysis, SUD

## Abstract

**Objectives:**

To conduct a socioeconomic analysis of Individual Placement and Support (IPS) integrated with specialized substance use disorder treatment. Additionally, we explored group differences in the use of social and welfare services and assessed the validity of a previous simulation model.

**Methods:**

We used Early Health Technology Assessment (eHTA) to estimate socioeconomic gain using data extracted from a completed randomized controlled trial comparing IPS and enhanced treatment as usual (enhanced TAU) for patients in specialized substance use disorder treatment.

**Results:**

IPS was socioeconomically beneficial compared to enhanced TAU during the first year after end of the intervention (€992,224 for IPS, and €778,121 for enhanced TAU), equal to a group difference of €214,103 (modelled for 100 patients). During the trial period, IPS participants showed a total cost saving of €157,593 due to less use of social and welfare services, compared to participants receiving enhanced TAU. These estimates are comparable to our previous eHTA simulation.

**Conclusion:**

Our estimates indicate a net socioeconomic gain of IPS starting during the intervention year, which exceeds that of enhanced TAU in the first year following the intervention. Additional cost savings were found in favor of IPS. Our early simulated eHTA proved valid in one scenario.

## Introduction

Patients with substance use disorders (SUDs) suffer a high personal burden with low quality of life, high prevalence of comorbid somatic and mental health problems, severe isolation, and early death [1, 2]. SUDs also represent a substantial economic burden for society with costs to social and healthcare services, reduced societal income due to loss of tax earnings and costs related to criminal justice services [3, 4]. Unemployment rates among SUD patients are high, and SUD treatment monitoring data from the UK report 72.9% of patients being unemployed [5]. Among patients admitted to SUD treatment in Norway, the estimated unemployment rate is 38.3% among those using legal substances and 54.4% among those using illegal substances [6]. Importantly, SUD patients themselves express a wish for work [7]. In addition to the obvious socioeconomic benefits of employment, it may also affect SUD recovery by protecting against relapse and facilitating community integration [8, 9]. Given the low labor market participation in this group, interventions beyond treatment may be needed to help individuals with SUD obtain and keep employment. Individual Placement and Support (IPS) is an evidence-based employment support method developed to help individuals with severe mental illness attain and keep a job in the competitive labour market. A key feature of IPS is that patients in treatment simultaneously receive individualized support from employment specialists who are fully integrated into the healthcare service, by being co-located, and working closely with the mental health practitioners [10]. Over 30 randomized controlled trials show that IPS is almost twice as effective as other vocational methods in supporting patients with moderate to severe mental disorders to obtain employment [11]. In recent years, the method has also proved promising for patients with SUDs [12–14]. The two most recent RCTs for patients with SUDs are the IPS-AD trial in the UK and the IPS-SUD trial in Norway [13, 14]. The IPS-AD trial compared IPS to treatment as usual (TAU) for SUD patients in treatment and found superior effects for the main outcome of attained employment, but no significant effects for any secondary vocational outcomes [13]. In the IPS-SUD trial, patients undergoing treatment for SUD received either IPS or enhanced TAU. Results showed a significant effect in favor of IPS for the secondary vocational outcomes of hours worked and salary [14].

There is considerable evidence showing cost-effectiveness and cost-benefit of well-implemented IPS services for users with severe mental illness (severe depression, bipolar and psychotic disorders) in high-income countries in Europe and North America [15]. However, the potential cost-effectiveness of IPS for SUDs is less known. The best available data come from the IPS-AD trial, where incremental cost-effectiveness ratios for quality-adjusted life years (QALY) were evaluated against willingness to pay (WTP) at two different thresholds, low (£30,000) and high (£70,000). IPS was found to be cost-effective only for the subgroups of patients with alcohol use disorder and other drug use disorders (excluding opiate use disorder) at the high WTP threshold (£70,000). For the opiate use disorder subgroup and the full sample of all SUDs, IPS was not found to be cost-effective at any of the WTP thresholds [13].

The cost of delivering IPS is high, given the long-term individual follow-up. When delivered from the health service, this cost impacts hospital budgets, while the financial gain of moving people off financial aid into paid work is shown on budgets outside of the health sector. Therefore, decisions related to implementation and organization of IPS should also be informed by cross-sector data regarding costs and benefits. To provide such data, a comprehensive analysis must incorporate socioeconomic benefits outside of traditional health economic outcomes.

In our recently conducted IPS-SUD trial [14, 16], four employment specialists delivered the IPS intervention, all employed by the hospital and integrated into the treatment teams. Before the trial, we conducted a socioeconomic simulation of IPS based on well-founded assumptions regarding its effects and costs drawn from Statistics Norway (SSB), hospital administration data, and expert opinion [17]. We assumed a 40% employment rate for participants receiving IPS and a 15% employment rate for participants receiving enhanced TAU. In the simulation study, we modeled three different scenarios based on the average proportion of full-time hours worked and duration of employment (working 100% and 50% of full-time hours for 10 years, and 25% of full-time hours for 5 years). This socioeconomic simulation showed an increased gain from IPS compared to enhanced TAU in all three scenarios. The simulation also showed that IPS would become socioeconomically beneficial sooner if participants worked more hours [17]. With the completion of the IPS-SUD trial, we now have updated data regarding its effects.

### Aims and objectives

The aim of this study was threefold: First, to conduct a socioeconomic analysis of Individual Placement and Support (IPS) integrated with specialized substance use disorder treatment. Second, to explore differences in the use and related costs of employment support services from the Norwegian Labour and Welfare Administration (NAV). Third, to assess the validity of a prior simulation study by updating the model with data from the IPS-SUD trial.

## Methods

This paper adheres to the Consolidated Health Economic Evaluation Reporting Standards [18]. We extracted the direct costs and vocational outcomes used for this socioeconomic analysis from unpublished and published data belonging to the IPS-SUD trial. The IPS-SUD trial was pre-registered at clinicaltrials.gov [Clinicaltrials.gov identifier, NCT04289415] and vocational outcomes from the trial have been published [14]. No specific analysis plan was developed for this socioeconomic analysis; however, it is an updated version of an early simulation model previously published [17].

### Description of the IPS-SUD trial

The IPS-SUD trial was a pragmatic randomized controlled trial implemented at Oslo University Hospital (OUH), Norway [16]. The participants (n = 187) were adults currently in specialized SUD treatment at OUH. They had a median age of 37 (IQR: 30–45), 22% were women, and the majority had been unemployed for 2 years or more (55%) [14].

The participants were recruited from five outpatient units and two residential units at OUH in the period from March 1st, 2020, to May 31st, 2022. Participants were randomized to receive either IPS or enhanced TAU. The IPS group was offered a maximum of 13 months of integrated employment support alongside their SUD treatment. The enhanced TAU group was offered a self-help guidebook, a three-session workshop in groups of no more than 13 participants, and an individual follow-up session with a group leader, alongside their SUD treatment. Because the IPS-SUD trial did not collect data for a TAU-only group, the current analysis of costs and benefits includes estimates only for the IPS and enhanced TAU groups, thus leaving out data on the TAU-only group, which was included in the previously published pre-trial socioeconomic simulation [17].

### Rationale and description of our socioeconomic model

The former simulation and the present study use Early Health Technology Assessment (eHTA) to assess the costs and socioeconomic effects of the intervention. eHTA is defined as a health technology assessment conducted to inform decisions about subsequent development, research, and/or investment by explicitly evaluating the potential value of a conceptual or actual health technology [19].

Since the cost of the IPS intervention is delivered by the hospital but its outcomes and savings impact on budgets outside the hospital, we chose to conduct a broad socioeconomic analysis, in contrast to a traditional health-economic analysis. Our economic model estimates effects in terms of direct costs of the intervention for the hospital, as well as its direct and indirect costs and effects for society. All costs and potential savings were reported in Norwegian Kroner (NOK) and converted to Euros using the exchange rate from January 29, 2025 (1 NOK = €0.0845). For modelling purposes, we scaled up each group to represent data for 100 participants over a 10-year time horizon.

### Direct costs

We estimated direct costs associated with the intervention from the payer’s perspective (i.e., the healthcare services), which included hospital charges and professional fees. The time horizon for the cost of the intervention was set to one treatment cycle of approximately 1 year; thus, the discount rate was 0%. IPS costs used in our primary analysis were informed by the total cost of having four full-time employment specialists delivering the intervention, in total €684,450. Costs of enhanced TAU were calculated for one person working 20% of full-time hours, based on full-time salary for one employment specialist position (€57,037).

Additionally, we included two sensitivity analyses. In the first we calculated costs of the intervention based on the number of hours of IPS delivery instead of using hospital charges and professional fees. We constructed two different scenarios: one using costs based on IPS intensity data extracted from the trial (10.4 h average IPS consultations per participant) and one using costs based on the assumptions of IPS intensity from the pre-trial simulation (87.5 h average IPS consultations per participant), multiplied by standard hourly rates sourced from SSB (€37). The sensitivity analyses also included a similar way to calculate enhanced TAU cost, which included costs of course coordination (1.5 h of work multiplied by an hourly rate of €30, divided by 13 participants) and course instruction (6 h multiplied by an hourly rate of €38, divided by 13 participants, including an additional hour of individual session per participant). All cost estimations were calculated for 100 participants in each group, giving a total of 1,040 h of IPS in our scenario based on trial data (IPS low intensive) and 8,750 h in the scenario based on pre-trial assumptions (IPS high intensive). The total patient contact for the sensitivity analysis of enhanced TAU (ETAU 2) was 158 h.

In our second sensitivity analysis we accounted for sample error from the RCT using probabilistic sensitivity analysis (PSA) [20]. We ran 2000 non-parametric bootstrap simulations with replacement for our input variables employment rate, working capacity and income for both the IPS and ETAU groups. The distribution of the simulated employment data can be found in Supplementary Material (Supplementary Table S2). All data related to costs and disability pension were identical to that of the main analysis and were set as fixed, due to them being based on external data only available as point estimates. Our socioeconomic analysis was then run on our simulated data distributions, which resulted in mean socioeconomic estimates including uncertainty in the form of 95% confidence intervals (95% CI).

Results from our PSA are shown in Supplementary Material (Supplementary Tables S1–S3).

As the intervention was financed through public budgets, we included a tax financing cost of 20 cents per euro (20%) in the model. This reflects the marginal cost of collecting an extra euro in taxes, as this may lead to inefficiencies. The estimated measure of 20% is based on the standard assumption in Norwegian public finance that a value creation on the private side of 20 cents is needed in order to finance one euro in a public project [21]. For a complete overview of resources and costs per participant, see Table 1; Supplementary Table S1.

**TABLE 1:** Cost specification of enhanced TAU (ETAU) and Individual Placement and Support (IPS), and total cost per participant’s one-year treatment cycle (in €), based on total salary (Norway, 2026).

Cost component	Calculation	Subtotal	Source
Enhanced TAU			
Course instruction + follow-up	€57,037.5/96 participants	€118.8	RCT
IPS			
Employment specialist	€684,450/91 participants	€7,521.5	RCT

### Indirect costs

We included indirect costs of production loss from disease and disability, which are estimated in terms of the intervention’s effect on disability pension and absence from the labor market. We assumed that patients who permanently exited the labor market after the intervention were expected to receive disability pension. The 2019 average annual disability pension of €21,379 per recipient [22], was used to show yearly effects of SUD patients excluded from the labor market. For patients who obtained employment, disability pension was reduced according to their working capacity. As disability pension is considered a social transfer payment, we only included the associated tax financing cost of the measure (€0.20 per €1.00) in our model.

### Direct effects

From the vocational data in the IPS-SUD trial [14] we used the employment rate for the two groups as the direct effect in our model. This input data was restricted to participants who achieved at least 3 months of paid employment, a threshold we considered a reasonable indicator of long-term employment and appropriate for the 10-year time horizon of our model. These employment rates were 29% among those who received IPS and 26% for those receiving enhanced TAU.

### Indirect effects

The average salary among those who achieved at least 3 months of paid employment was €22,841 in the IPS group and €10,762 in the enhanced TAU group. The salary included employer’s contribution and social costs, used for value creation per year for a person who entered the labor market after the intervention. We assumed an income tax of 25% and an employer’s contribution of 14.1% in our calculations of increased tax revenue [23].

These future benefits were discounted at a rate of 4%, which is in line with recommendations from the Norwegian Ministry of Finance on public investment projects [24].

### Intervention effects on self-reported use of employment support services from NAV

The second objective for the present study was to evaluate the impact of IPS on the participants' use of employment support services administered by the Norwegian Labour and Welfare Administration (NAV). In this separate analysis, we extracted the self-report data for those participants in our trial who responded at 6 months follow-up (n = 123) and/or 12 months follow-up (n = 122). Some participants received several services. Different employment support services were categorized as: employment preparation training, employment training, IPS, work capacity assessment, and “other.” Twice as many in the enhanced TAU group reported using an employment support service from NAV, compared to the IPS group (18/58 vs. 9/64). The difference in proportion of employment support service use in the enhanced TAU group and the IPS group was multiplied by the cost associated with each service and summarized as a cost-difference in Table 3. The cost associated with the different employment support services was based on a report prepared for the Ministry of Labour and Social Inclusion for the first three services, and the fourth service, work capacity assessment, was based on expert opinion [25]. We did not manage to price a fifth category, “Other”.

## Results

### Socioeconomic effect

**FIGURE 1:**
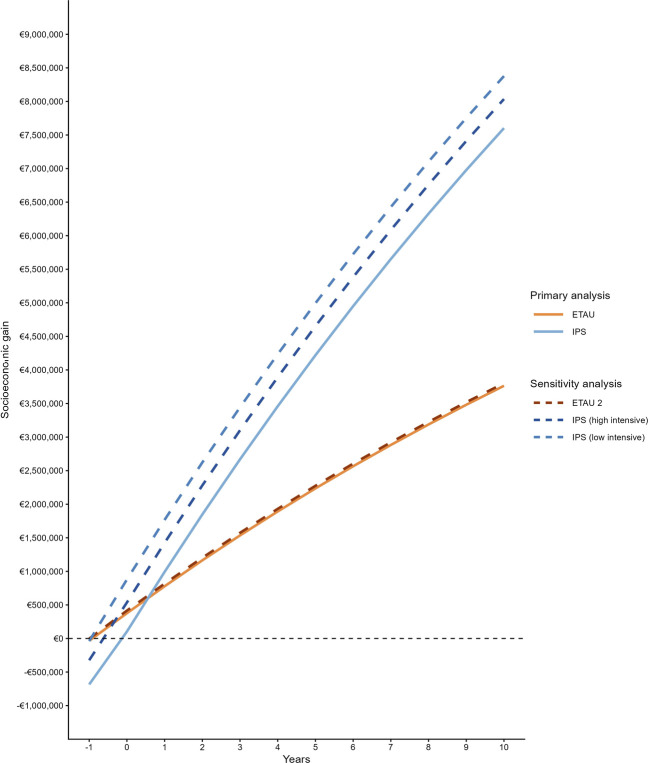
Estimated socioeconomic gain for Individual Placement and Support (IPS) and enhanced TAU (ETAU). The model projects results for a cohort of 100 participants in each group over a 10-year period. Solid lines represent the primary analysis (intervention costs based on total salary), while dashed lines represent the sensitivity analysis (costs based on direct patient contact hours). The horizontal dotted line represents the break-even point (Norway, 2026).

A summary of socioeconomic effects per participant at 10 years is shown in Table 2, with the potential socioeconomic gain of implementing IPS for 100 SUD patients over 10 years illustrated in Figure 1. Given the assumptions in the model, scaled for 100 participants in each group, the socioeconomic value of IPS was estimated to exceed enhanced TAU during the first year (year 0–1) after end of the intervention (€992,224 for IPS, and €778,121 for enhanced TAU), equal to a difference of €214,103. The 5-year estimates were €4,219,207 for IPS and €2,235,751 for enhanced TAU. The 10-year estimates had increased to a gain of €7,602,247 for IPS and €3,763,872 for enhanced TAU, with a difference of €3,838,375.

**TABLE 2:** Socioeconomic analysis of enhanced TAU and Individual Placement and Support (IPS) showing the cost (in €) of 1 year of the intervention and outcomes in terms of social effects (in €) per participant 10 years after end of intervention (Norway, 2026).

Costs and effects	Enhanced TAU	IPS
Costs associated with the intervention groups	119	7,522
Increased value creation^1^	81,709	173,799
Impact on public budgets^1^	22 322	31,073
Socioeconomic effect^1^	103 912	196,970

1 Based on average income among participants for each group obtaining 3 months of employment (25/96 [26%] in enhanced TAU, and 26/91 [29%] in IPS).

In the sensitivity analysis based on hourly delivery (low vs. high intensive), the socioeconomic value estimates from both IPS scenarios were expected to surpass enhanced TAU (ETAU 2) during the intervention year (year −1 to 0) following the intervention start (Enhanced TAU = €415,101; IPS low intensive = €878,384; IPS high intensive = €536,058).

In the PSA, the re-estimated point estimates for the three employment variables were quite similar to those in the main analysis (Supplementary Table S2). The resulting socioeconomic analysis based on these numbers show similar trends as the main analysis. The socioeconomic value estimate of IPS surpasses ETAU during first year after intervention with a mean difference of 324,960 (Supplementary Figure S1). However, the PSA showed large uncertainties in this point estimate (95% CI: −462,357 to 1,124,683). In case of best scenario (upper bound of the 95% CI) for both groups, net gain of IPS occurs within the intervention year, while in a worst case scenario (lower bound of the 95% CI) for both groups, a net gain of IPS occurred within the second year after intervention end (year 1–2). However, in case of worst case performance of IPS compared to best case ETAU, IPS does not surpass ETAU (as illustrated by overlapping CIs).

The yearly socioeconomic gain, including mean differences between each group and a graph depicting them, can be found in Supplementary Material (Supplementary Table S3; Supplementary Figure S1).

### Intervention effects on self-reported use of employment support services from NAV

Table 3 shows the proportion of employment support services used in the IPS group and the enhanced TAU group, the cost per participant for each service, and the cost difference between the two groups. In total, the IPS participants had less service use in four out of five employment support service categories, totaling a group cost saving of €157,593 relative to the participants receiving enhanced TAU.

**TABLE 3:** Self-reported use of employment support services administered by the Norwegian Labour and Welfare Administration and the associated average cost (in €) per service and cost difference between the groups, among services reported by those who participated in 6 and/or 12 months follow-up (n = 147) (Norway, 2026).

Type of service	Enhanced TAU	IPS	Cost per participant	Total cost difference (group)
Any employment support service	33	15	​	157,593
Employment preparation training	11	3	20,618	164,944
Employment training by NAV	8	3	761	3,805
Individual placement and support	3	7	4,648	−18,592
Work capacity assessment	4	0	1,859	7,436
Other	7	2	N/A	​

### Comparing current updated model to earlier simulated model

In this updated eHTA, the estimated socioeconomic gain of IPS was found to exceed that of enhanced TAU during the first year after the intervention, and this change was observed 1 year later than in the pre-trial eHTA. Our sensitivity analysis of costs estimated IPS to exceed enhanced TAU already during the intervention year, which is similar to the pre-trial eHTA. Our sensitivity analysis using PSA gave the same conclusion as the main analysis, but showed that the mean difference estimate has large uncertainty (Supplementary Table S3).

## Discussion

In this socioeconomic analysis of IPS vs. enhanced TAU for patients in SUD treatment, we found an estimated socioeconomic gain in favor of IPS within the first year after end of intervention, increasing towards year 10 (Figure 1). In our separate analysis of employment support usage, the enhanced TAU group used more than twice the amount of employment support services from NAV, as compared to the IPS group. In this updated eHTA, we found IPS to have a higher cost, employment rates to be more balanced between the groups, and participants receiving IPS working more hours and earning more than we assumed in the pre-RCT eHTA.

Our primary analysis indicates that despite IPS being a resource-intensive investment per participant (€7,521), its socioeconomic net benefit reached zero (breakeven) during the intervention year, and our PSA indicating this breakeven may happen 1 year later, after the end of intervention. Further, as compared to investing in the enhanced TAU intervention, investing in IPS within the hospital is potentially beneficial during first year after the intervention according to our main analysis. However, our PSA cautions this conclusion showing large uncertainties in our point estimates (Supplementary Table S3). The socioeconomic benefits of IPS increased beyond the first year; however, these long-term estimates should be considered with caution due to high uncertainty regarding the duration of employment and parameter uncertainty.

The findings from the RCT that the enhanced TAU group was significantly more likely to receive services from NAV [14] were further explored by estimating the costs of the services confirmed used during the trial based on the participants’ self-report. This result indicated a substantial economic impact (€157,593). This shows that employment support in the health service alleviates the service burden for NAV. It may be that since all participants who were recruited to the trial were motivated to work, those who were not randomized to receive employment support (IPS) as part of their treatment may simply have sought such support from NAV, thus explaining this difference. Also, the difference may be an effect of the enhanced TAU intervention, which aimed at providing information and support on how to best make use of the employment support services at NAV. Of course, the exact effect of the enhanced TAU intervention would be best evaluated by comparing it directly to TAU. However, our results show the enhanced TAU only intervention as being low cost, easy to implement, and potentially efficient.

Comparing the previous and current updated eHTA, we underestimated the costs of IPS in the early pre-trial eHTA (€3,813 compared to €7,521) largely driven by the differences in calculating costs, as we in this eHTA calculated costs in a more conservative way based on the total salary of four employment specialists. In our sensitivity analysis, we kept the pre-trial eHTA cost assumptions regarding IPS intensity (87.5 h per participant) and included a new one based on the extracted data from the RCT (10.4 h per participant). For both these scenarios, the gain of IPS exceeded enhanced TAU during the first year after intervention, similar to that of the previous eHTA. Further, we underestimated the employment rate for the enhanced TAU group, which turned out to be substantially higher (34.4% with at least 1 day of employment, 26% with 3 months or more) than we assumed pre-RCT (15% with at least 1 day of employment). These differences resulted in a substantially higher cost of the IPS intervention and a larger benefit of enhanced TAU, giving a smaller overall difference between the two groups in terms of socioeconomic gain as compared with the estimates from the early eHTA simulation model.

The previous eHTA had three simulated scenarios, and compared to our updated eHTA, in which 29% of IPS participants worked 68% of full-time hours and 26% of ETAU participants worked 50% of full-time hours, it fits best between the balanced scenario (40% vs. 15% of participants holding a half-time position over 10 years), and the conservative scenario (40% vs. 15% of participants working full-time positions for 10 years). Due to this updated eHTA not including costs of SUD treatment, the specific net estimates are not directly comparable to the previous eHTA.

Norway is known for its extensive welfare system with relatively high social and disability benefit payouts and high levels of recipiency as compared to other OECD countries [26]. Countries with high social and disability benefit payouts have been described as at risk of creating “benefit traps”, functioning as economic disincentives for obtaining employment in terms of, for instance, losing housing benefits or reduced overall income [27]. Meta-analytic evidence from IPS trials suggests a lower employment rate in countries where benefits are perceived to be higher than the overall income from employment (a larger risk of a benefit trap), independent of the comparator intervention [27]. This may have impacted the outcomes of our RCT and hence the results of this eHTA. Further, the effect of IPS has also been found to be reduced in areas where local employment rates are substantially lower than national rates [27]. Since our trial was conducted in the capital Oslo, which has a somewhat higher unemployment rate compared to national rates, one can assume that the effect of IPS would be somewhat better in, for instance Northern Norway, an area with a substantially lower unemployment rate compared to national rates [28].

The trial took place during the COVID-19 pandemic, including two long lockdowns. The multiple ways this may have impacted on our trial is discussed in our previous publication from the IPS-SUD trial [14]. The pandemic impacted the labor market in general and strict social distancing policies were present particularly in Oslo, the capital of Norway. This impacted our employment specialists ability to meet face-to-face with potential employers and patients. COVID may therefore have had a disproportionate effect on the IPS arm in the trial, given that one-to-one meetings with patients and potential employers is a core component of the IPS intervention. Further, we are uncertain whether the relatively low degree of service use (average participant having 10.4 h) captures the true extent of contact between participants and their employment specialists, as the shift towards digital and phone-based forms of contact occurred rapidly, and these new types of contact may have been insufficiently registered in the hospital systems.

### Limitations

Our results should be interpreted with some caveats. First, the extrapolation of study results over 10 years represents an early assessment of potential benefits; thus, the results are merely formative and should be reassessed when more long-term data is available. Second, we chose to extrapolate the employment input only from participants with employment exceeding 3 months, resulting in a small dataset on salary and FTEs in both groups. Thus, there is uncertainty in the amount and time horizon of the cost and potential savings estimates. Third, though our analysis of costs and benefits includes information from health and social welfare services, it lacks information from the criminal justice sector, and it does not include potential new treatment contacts or rehospitalizations from the health sector. It may be assumed that employment is associated with reduced crime and reduced risk of rehospitalization, meaning that if these factors were included, the estimates would potentially show an even larger gain from IPS. Fourth, the generalizability of our results may be most relevant to countries with comparable welfare systems. Lastly, our analysis is based on the effect of IPS during strict periods of social distancing due to the COVID-19 pandemic, which may have disproportionately impacted the IPS group and thereby reduced the socioeconomic impact of IPS and also the generalizability of our results.

### Conclusions

Our updated eHTA model estimates that IPS, despite being modeled with a conservative high cost, achieves a greater net socioeconomic gain during the first year after the end of the intervention, compared to enhanced TAU. The conservative scenario of our earlier eHTA, which was based on registry data and expert opinions pre-RCT, provided valid estimates comparable with this updated eHTA based on post-RCT data. Compared with the enhanced TAU group, the IPS group showed cost savings due to less use of services from NAV. Our socioeconomic analysis based on costs and benefits from budgets across different sectors, can provide valuable information on the societal effect of IPS, supplementing estimates resulting from traditional health-economic analysis. From the perspective of decision-makers, it will be informative to consider the overall socioeconomic effect when assessing the organization and implementation of IPS, especially since IPS directly impacts beyond hospital budgets. Future research on the long-term duration of employment for this patient group would be an important next step to provide better data on estimating socioeconomic gain.

## Data Availability

The full dataset for this article is not publicly available due to ethical restrictions regarding patient anonymity and the sensitive nature of the data. Requests to access the datasets should be directed to the corresponding author, and data can only be shared after permission from the ethics committee and institutional review board.
