# Supplementary material for: Individual placement and support (IPS) integrated with specialized substance use disorder treatment: a socioeconomic analysis based on a randomized controlled trial
Source: Int J Public Health. 2026 Jun 25;71:1609386. doi: 10.3389/ijph.2026.1609386 (PMC13345976; doi:10.3389/ijph.2026.1609386)

Supplementary Figure S1. Estimated socioeconomic gain for Individual Placement and Support (IPS) and enhanced TAU (ETAU) based on the probabilistic sensitivity analysis. The model projects the result for a cohort of 100 participants in each group over a 10-year period. The horizontal dotted line represents the break-even point. The faded color above and below the lines area represents the corresponding 95% Confidence Intervals.


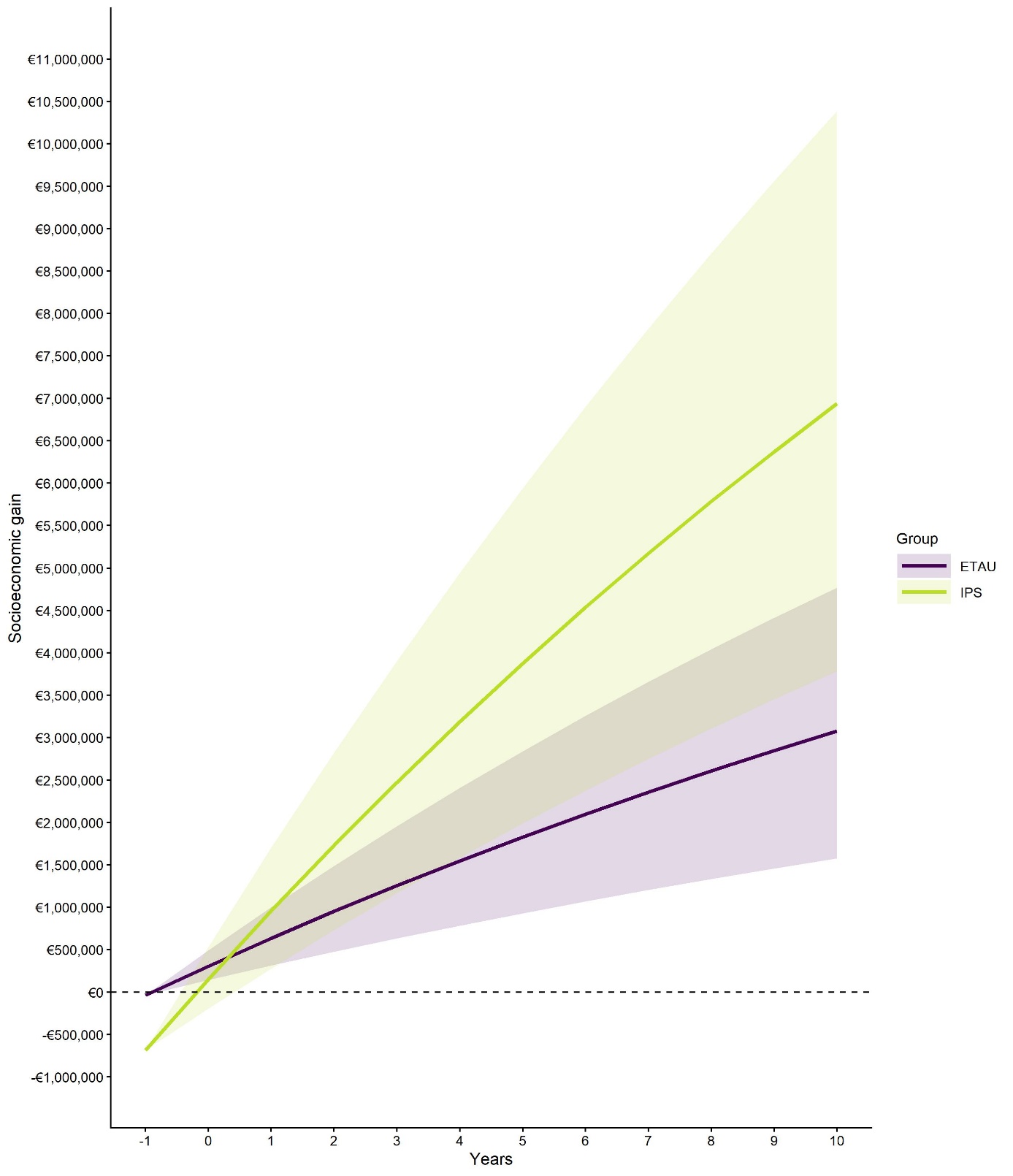

Supplement: Supplementary file 1 [file Supplementaryfile1.docx]
