# Supplementary material for: Individual placement and support (IPS) integrated with specialized substance use disorder treatment: a socioeconomic analysis based on a randomized controlled trial
Source: Int J Public Health. 2026 Jun 25;71:1609386. doi: 10.3389/ijph.2026.1609386 (PMC13345976; doi:10.3389/ijph.2026.1609386)
Supplement: Supplementary file 2 [file Table1.docx]

| Supplementary Table S1. Sensitivity analysis (based on costs) cost specification of enhanced TAU (ETAU2) and Individual Placement and Support (IPS) based on IPS intensity data. | | | |
| --- | --- | --- | --- |
| Cost component | **Calculation** | **Subtotal** | **Source** |
| ETAU 2 |  |  |  |
| Course Instruction +  Follow-up | [(6 hours) × €38/hour] /13 participants + 1 Hour | 55.5 | RCT,  Statistics Norway |
| Course Coordination | (1.5 hours × €30/hour)  /13 participants | 3.5 |  |
| Total Cost ETAU 2 |  | 59 |  |
| IPS (low intensive) |  |  |  |
| Job Specialist  Consultations | 10.4 hours × €37/hour | 384.8 | RCT,  Statistics Norway |
| IPS (high intensive) |  |  |  |
| Job Specialist  Consultations | 87.5 hours × €37/hour | 3,237.5 | Pre-trial assumptions,  Statistics Norway |
