# Supplementary material for: Individual placement and support (IPS) integrated with specialized substance use disorder treatment: a socioeconomic analysis based on a randomized controlled trial
Source: Int J Public Health. 2026 Jun 25;71:1609386. doi: 10.3389/ijph.2026.1609386 (PMC13345976; doi:10.3389/ijph.2026.1609386)
Supplement: Supplementary file 3 [file Table2.docx]

| **Supplementary Table S2.** Simulated data (N=2000) for the employment variables used as model input in sensitivity analysis (employment rate, income and working capacity) | | | |
| --- | --- | --- | --- |
| IPS | Mean (SD) | Min-max | Median (IQR) |
| Employment rate (%) | 28 (5) | 15-47 | 29 (25-32) |
| Working capacity (%) | 68 (6) | 45-89 | 68 (64-72) |
| Income (€) | 22,936 (3,148) | 14,266 - 33,941 | 22,933 (20,875 - 24,978) |
| ETAU |  |  |  |
| Employment rate (%) | 26 (5) | 11-40 | 26 (23-29) |
| Working capacity (%) | 50 (7) | 23-74 | 50 (46-55) |
| Income (€) | 10,760 (1,790) | 4,141- 16,544 | 10,737 (9,520 – 11,937) |
