# Supplementary material for: Individual placement and support (IPS) integrated with specialized substance use disorder treatment: a socioeconomic analysis based on a randomized controlled trial
Source: Int J Public Health. 2026 Jun 25;71:1609386. doi: 10.3389/ijph.2026.1609386 (PMC13345976; doi:10.3389/ijph.2026.1609386)
Supplement: Supplementary file 4 [file Table3.docx]

| Supplementary Table S3. Probabilistic Sensitivity Analysis estimates for each intervention (in €), including mean difference with corresponding 95% Confidence Intervals. | | | |
| --- | --- | --- | --- |
| Time | Mean IPS (95% CI) | Mean ETAU (95% CI) | Mean Diff (95% CI) |
| -1 | -684,450 (-684,450 to -684,450) | -34,222 (-34,222 to -34,222) | -650,228 (-650,228 to -650,228) |
| 0 | 156,399 (-190,967 to 534,062 | 309,472 (138,730 to 500,544) | -153,073 (-554,451 to 254,629) |
| 1 | 964,909 (283,536 to 1,705,708) | 639,948 (305,030 to 1,014,743) | 324,960 (-462,357 to 1,124,683) |
| 2 | 1,742,321 (739,789 to 2,832,291) | 957,713 (464,934 to 1,509,164) | 784,608 (-373,806 to 1,961,274) |
| 3 | 2,489,833 (1,178,494 to 3,915,544) | 1,263,257 (618,687 to 1,984,570) | 1,226,577 (-288,661 to 2,765,688) |
| 4 | 3,208,595 (1,600,325 to 4,957,133) | 1,557,049 (766,528 to 2,441,690) | 1,651,546 (-206,790 to 3,539,163) |
| 5 | 3,899,712 (2,005,932 to 5,958,661) | 1,839,541 (908,682 to 2,881,229) | 2,060,171 (-128,069 to 4,282,889) |
| 6 | 4,564,248 (2,395,939 to 6,921,669) | 2,111,168 (1,045,368 to 3,303,863) | 2,453,080 (-52,375 to 4,998,010) |
| 7 | 5,203,224 (2,770,946 to 7,847,638) | 2,372,348 (1,176,798 to 3,710,242) | 2,830,877 (20,408 to 5,685,627) |
| 8 | 5,817,625 (3,131,529 to 8,737,993) | 2,623,482 (1,303,172 to 4,100,990) | 3,194,143 (90,391 to 6,346,796) |
| 9 | 6,408,394 (3,478,243 to 9,594,104) | 2,864,958 (1,424,686 to 4,476,710) | 3,543,437 (157,683 to 6,982,536) |
| 10 | 6,976,442 (3,811,623 to 10,417,287) | 3,097,145 (1,541,526 to 4,837,979) | 3,879,297 (222,386 to 7,593,825) |
